# Fatty Acids in Energy Metabolism of the Central Nervous System

**DOI:** 10.1155/2014/472459

**Published:** 2014-05-04

**Authors:** Alexander Panov, Zulfiya Orynbayeva, Valentin Vavilin, Vyacheslav Lyakhovich

**Affiliations:** ^1^Institute of Molecular Biology and Biophysics, Siberian Division of the Russian Academy of Medical Sciences (SB RAMS), 2 Timakova st., Novosibirsk 630117, Russia; ^2^Department of Surgery, Drexel University College of Medicine, Philadelphia, PA, USA

## Abstract

In this review, we analyze the current hypotheses regarding energy metabolism in the neurons and astroglia. Recently, it was shown that up to 20% of the total brain's energy is provided by mitochondrial oxidation of fatty acids. However, the existing hypotheses consider glucose, or its derivative lactate, as the only main energy substrate for the brain. Astroglia metabolically supports the neurons by providing lactate as a substrate for neuronal mitochondria. In addition, a significant amount of neuromediators, glutamate and GABA, is transported into neurons and also serves as substrates for mitochondria. Thus, neuronal mitochondria may simultaneously oxidize several substrates. Astrocytes have to replenish the pool of neuromediators by synthesis de novo, which requires large amounts of energy. In this review, we made an attempt to reconcile *β*-oxidation of fatty acids by astrocytic mitochondria with the existing hypothesis on regulation of aerobic glycolysis. We suggest that, under condition of neuronal excitation, both metabolic pathways may exist simultaneously. We provide experimental evidence that isolated neuronal mitochondria may oxidize palmitoyl carnitine in the presence of other mitochondrial substrates. We also suggest that variations in the brain mitochondrial metabolic phenotype may be associated with different mtDNA haplogroups.

## 1. Introduction

The architecture of the body and the structure of enzymes, which determine the body's functions, are encoded in the nuclear (nDNA) and mitochondrial (mtDNA) DNA. All functions, including replication of DNA and synthesis of enzymes, require energy provided in higher organisms by mitochondria. Thus, life involves the interplay between structure and energy [1].

Until recent centuries, the indigenous populations of the Earth lived sedentary for thousands of years in different climate zones and thus were forced to adapt to different types of food and temperatures. Mitochondria play the leading role in adaptation of animals and humans to environmental conditions to a large degree due to high mutation rate of mtDNA [1]. Rapid segregation of variant mtDNAs within the female germline results in maternal lineages close to homoplasmic mtDNAs [2]. Wallace stressed that individuals harboring variant mtDNA genotypes differ in mitochondrial physiologies and thus will respond differently to changing environmental and pathological conditions [3]. This led to significant variations of mtDNA in indigenous populations from different parts of the world. As a result, the human mtDNA tree has discrete branches with each branch including a group of related mtDNA sequences (haplotypes) called a haplogroup [4]. The haplogroups correlate with the geographic distribution of indigenous populations and consequently with their environmental niche, and, moreover, they have different predisposition to various pathological conditions [4–8]. The mutation rate of mtDNA is high not only in the female germline but also in somatic tissues of the body. Consequently, mtDNA rearrangement and base substitution have been found to accumulate with age in multiple tissues [1, 3].

In contrast to the impressive achievements of molecular biologists in the study of mtDNA and association of variations and mutations of mtDNA with pathology, which were depicted in several fundamental reviews by one of the “fathers” of Mitochondrial Medicine, Wallace, investigations of mitochondrial physiology and biochemistry failed to give clear answers on the mechanisms that underlie connections between mtDNA haplogroups and pathology [1, 3, 4]. Also, an important fundamental question is why, in patients with systemic distribution of mutated mtDNA, clinical manifestations are usually organ-specific and different mutations have different clinical pictures.

In a number of recent publications, we have stated that the most common or traditional methods of studying mitochondrial functions are outdated and even obsolete, if the goals of the studies are mitochondrial physiology and pathophysiology [9–11]. It is clear, that* in situ* mitochondria oxidize not a single substrate but a mixture of substrates depending on the metabolic situation, hormonal status, and type of the host cell. However, in most publications on mitochondrial functions, the authors used only a single substrate or substrate mixture glutamate + malate and pyruvate + malate. In too many papers, succinate + rotenone was used as the only substrate, which is completely unacceptable. There is also a popular belief that glutamate and pyruvate are “classical” substrates for complex I, while succinate is substrate for complex II. However, we have shown that, in brain and spinal cord mitochondria, up to 50% of added glutamate or pyruvate was metabolized via transamination to *α*-ketoglutarate and then converted to succinate [10]. Some researchers still believe that there is no major difference between mitochondria from various organs and consider liver mitochondria as a “standard” for mitochondrial functions.

In addition, a sharp drop in publications describing mitochondrial functions, respiration in particular, during the last decade was probably associated with the ubiquitous usage of Milli-Q water for preparation of buffers. The Milli-Q water contains very little amount of cations and anions but is highly contaminated with peroxides [11]. Plastic containers and filter sterilization of buffers also contribute to significant contamination of experimental solutions with peroxides [11]. Thus, a complex mixture of circumstances and causes was responsible for inability of researchers studying mitochondrial physiology and biochemistry to match the advances in the mitochondrial molecular biology. The technical issues were discussed recently in [11, 12].

In this review we discuss facts and ideas available in the literature regarding the energy metabolism of mature brain in order to better understand mitochondrial functions in CNS. We formulate a new methodological approach to the brain's energy metabolism and neuronal mitochondria: both astroglial and neuronal mitochondria oxidize not a single substrate but a mixture of substrates, which include *β*-oxidation of fatty acids, pyruvate, lactate, and neuromediators glutamate and GABA. This will lead us to a better understanding on the roles of metabolic phenotypes, which are physiological expressions of the variant haplogroups of mtDNA and variant nDNA, in the development of metabolic and neurological diseases.

## 2. General Considerations Regarding Mitochondrial Metabolism

All cellular functions in higher animals require energy in the form of ATP, NADPH, ΔpH, and ΔΨ across the membranes and concentration gradients of metabolites, all of which are generated directly or indirectly by mitochondria. Mitochondria are organelles, which produce “useful” energy by utilizing part of the free energy released during the burning of hydrogen derived from different food sources: carbohydrates (glucose), fats (fatty acids), and proteins (amino acids). This presumes that the basic principles of mitochondrial functions have to be the same in all organs and tissues, but as we will see in the examples of neurons and astrocytes, the “details” and “variations” are very important for adaptation of mitochondrial metabolism to the specific functions of organs and cell specialization.

Since mitochondria produce energy by burning hydrogen, it is useful to appraise the hydrogen stores in the body taking into consideration functional and metabolic specialties of different organs. Let us first reckon carbohydrates. The content of glucose in the blood is close to 1000 mg per 1 L, that is, 5 grams in an average person with 5 liters of blood. This amount of glucose will be consumed by erythrocytes alone just in 20–30 minutes, and, in a much lesser time, if glucose is also consumed by the brain, spinal cord, heart, skeletal muscles, kidneys, and liver. The largest storage of glucose in the liver as glycogen is about 100–120 gram. Only glucose from the liver's glycogen can be shared with other organs [13]. We should also keep in mind that fat tissue also consumes large amount of glucose. This is because, in the fat cells, there is a constant cycling of fatty acids: first, triglycerides split into free fatty acids and glycerol, and then the same fatty acids become reconnected to a new glycerol forming a “new” triglyceride, while the “old” glycerol is catabolized. As a result, after about 2-3 hours of mild work, all reserves of carbohydrates (glucose) will be depleted. The constant level of glucose in the blood must be maintained for the sake of normal functioning of cells, which have absolute requirement for glucose: erythrocytes, nerve tissue, and fat tissue. Therefore, in order to maintain the physiologically constant level of glucose in the blood, the liver metabolism must switch early from glycolysis to gluconeogenesis.

The total storage of amino acids is very small, and, during gluconeogenesis and neuronal activity, the pool of amino acids must be constantly replenished by transamination of *α*-ketoglutarate, pyruvate, or proteolysis of proteins. Fat is the largest and most energetically rich and long lasting storage of hydrogen.

Thus, the constantly hard working organs, such as heart and kidneys, must utilize fatty acids as the major source of hydrogen. There is a saying which states that “fats are burned in the flame of carbohydrates.” In small animals and in some human phenotypes (northern indigenous populations), liver must produce glucose almost constantly. It is known that functions of the isolated liver mitochondria from experimental animals are highly dependent on the metabolic state of the liver [11, 14, 15].

From the common sense, it seems logical that mitochondria* in situ* never oxidize a single substrate: electrons enter the respiratory chain at different sites and from different metabolic pathways. Thus, the scientifically sound method to study mitochondrial functions suggests that a researcher must utilize the physiologically relevant mixtures of substrates, which may be different for different tissues. However, this issue has not yet been thoroughly studied. Although physiologists had known for a long time that heart, for example, utilizes for energy production a mixture of fatty acids and glucose, researchers, when studying isolated heart mitochondria, continue utilizing succinate + rotenone or glutamate, which is not a physiological substrate for the heart. We have stressed recently on the significance of physiologically relevant substrate mixtures to study the brain and spinal cord mitochondria functions [10, 16].

## 3. The Role of the Blood Brain Barrier in Regulation of the Brain Energy Metabolism

Unlike liver mitochondria, the respiratory activities of the isolated brain and spinal cord mitochondria do not depend much on the metabolic state of experimental animals due to the presence of the blood brain barrier (BBB) [17]. BBB is formed by the endothelial cells of the blood capillaries [18–20] and astroglial cells [21] that line cerebral microvessels. The combined surface area of these microvessels constitutes the largest interface for the blood-brain exchange. This surface area, depending on the anatomical region, is between 150 and 200 cm^2^ × g^−1^ tissue giving a total area for exchange in the brain between 12–18 m^2^ for the average human adult [22]. The processes of astrocytes form a virtually continuous sheath around the vascular walls; only about 11% of the vessel perimeter lack this astrocytic glia covering [21]. Neurons, glia, and microvessels are organized into well-structured neurovascular units, which regulate cerebral blood flow and maintain a precisely regulated microenvironment for reliable neuronal signalling [17, 18, 20]. BBB forms a barrier because tight junctions between adjacent endothelial cells force most molecular traffic to occur across the BBB through the cells [17]. Gases, O_2_, CO_2,_ and NH_3_, can freely diffuse through the lipid membranes, and the unbound long-chain fatty acids also diffuse through the membranes [23]. For most other compounds, including ions, there are specific transport systems. Finally, a combination of intracellular and extracellular enzymes provides a “metabolic barrier,” which can metabolize and inactivate many neuroactive and toxic compounds [17, 24]. However, the independence of the brain from the whole body metabolism is relative. In fact, hypothalamus has a rather easy access for many compounds in the blood, for example, carnitine and long-chain fatty acids, through numerous “fenestrations” in the BBB [23–25]. The hypothalamus has regions where the capillary endothelium is channeled to allow free passage of even large proteins and other molecules such as peptide hormones, whereas other sites serve as sensors of nutrients in the blood [23, 26]. There is a hypothesis that fatty acids metabolism within discrete regions of hypothalamus functions as a sensor of nutrient availability that participate in the integration of the energy balance via controlling multiple nutritional and hormonal signals [23]. Other brain sites did not show variance for glucose and fatty acid metabolism relative to feeding status [17].

## 4. The Problem of the Energy Substrate in the Brain

Brain functions are absolutely dependent on the aerobic mitochondrial energy metabolism. Individual neurons are rarely more than 8–20 *μ*m away from a brain capillary [27]. Oxygen is better soluble in lipids than in water; therefore, the membranous structures of the BBB are not a barrier for O_2_. The short distance from the blood capillaries ensures that neurons obtain enough oxygen for mitochondrial respiration. To this day, most researchers accept that “glucose is the only blood-borne substrate used by the normal, adult brain that can support its metabolic demands” [28]. The dissensions consider only what form neurons use glucose as a source of energy: as glucose [29, 30] or as lactate [31–39]. Pellerin and Magistretti have discovered the glutamate-stimulated aerobic glycolysis in astrocytes and that lactate, after conversion to pyruvate, serves as the substrate for mitochondrial respiration in activated neurons [32–39]. The concept is known as the astrocyte-neuron lactate shuttle hypothesis (ANLSH) [38, 40, 41].

In a recent comprehensive review, Bouzier-Sore and Pellerin [42] summarized the latest publications and analyzed the results of different approaches regarding utilization of glucose and lactate by neurons and astrocytes. The results of genetic and biochemical studies, both* in vivo* and* in vitro*, using nuclear magnetic resonance (NMR) spectroscopy of the ^1^H- and ^13^C-labeled glucose, lactate, and other metabolites, have unequivocally led to conclusion that most of the glucose is utilized by astrocytes providing neurons with lactate as a source of energy (see [42] for references and discussion). However, other researchers expressed objections and asked important questions that called for alternative hypotheses on the brain's energy metabolism [28, 43, 44]. The dissensions considered mostly the metabolism of astrocytes. Dienel and Hertz, despite admitting that lactate can be a good fuel for the brain cells under certain conditions, argued that the available evidence suggests that, when large amounts of lactate were formed, lactate was quickly cleared both from the cells and from the region of activated tissue, where it was generated to be consumed elsewhere [28]. Based on a series of studies, Dienel and Hertz suggested that (i) the activity of oxidative pathways increases in working astrocytes* in vivo* and* in vitro*, (ii) oxidative pathways produce two to three times more ATP than glycolytic lactate production during exposure of cultured astrocytes to 100 mM glutamate, and (iii) glutamate accumulated in the astrocyte can enter the TCA cycle and serve as a substrate for mitochondrial ATP production [28]. The latter hypothesis was quickly abandoned because it was discovered that, in astrocytic mitochondria, the content of the glutamate-aspartate transporter (GAT) is very low [45, 46]. Dienel and Cruz have noted that the energetic demands of activated astrocytes were higher and more complex than recognized [43]. In addition,* in vivo* activation studies revealed that the rise in consumption of the blood-borne glucose usually exceeded that of oxygen [43]. This mismatch between glucose and oxygen utilization becomes particularly large if, to consider the contribution of glycogen, the brain's major glucose reserve is located in astrocytes.

The failure of local oxygen consumption to become equal to that of glucose plus glycogen* in vivo* was considered as strong evidence against stoichiometric transfer of lactate from astrocytes to neighboring neurons for oxidation [43]. Dienel and Cruz suggested that astrocytes, not nearby neurons, use the glycogen and glycolysis for energy during physiological activation in normal brain [43]. They have also admitted that tissue culture studies do not consistently support the lactate shuttle hypothesis because key elements of the model, glutamate-induced increases in glucose utilization, and lactate release are not observed in many astrocyte preparations [43]. This brushed away many objections against the astrocyte-neuron lactate shuttle (ANLS) model. Here, we have to add that in many published experiments, the cells were cultured in the presence of penicillin + streptomycin (see, e.g., [47–49]). Mitochondria isolated from cultured cells grown in the presence of streptomycin have no or very low respiratory activity [50]. Therefore, the results obtained with cultured cells, grown in the presence of streptomycin, must be treated with caution.

## 5. Alternative to Glucose Substrates for the Brain Energy Metabolism

It has long been known that fatty acids can enter the brain [51, 52] and be oxidized [53–55]. It was estimated recently that about 20% of the total energy expenses of the adult brain are satisfied by oxidation of fatty acids [56]. It is believed that fatty acid oxidation occurs almost exclusively in astrocytes [56], and BBB is capable to transport carnitine and fatty acids from the blood to astrocytes [17, 57]. However, even in the latest discussions of the brain's metabolism [41, 42, 44, 58, 59], the role of fatty acids as important energy substrates for the brain energy metabolism was not taken into consideration. Although some researchers accept that the neuromediators glutamate [60] and GABA [61] may undergo oxidative degradation both in neurons and astroglia, there was little evidence obtained at the mitochondrial level. We have recently presented the evidence that isolated brain and spinal cord mitochondria require amino acid glutamate for the maximal rates of oxidative phosphorylation [10, 62]. Thus the energy metabolism of the central nervous system is much more complex and compartmentalized than it was thought.

## 6. Mitochondrial Metabolism and Functions Depend on Localization within the Cells

In order to understand mitochondrial metabolism in the brain and spinal cord, we have to take into consideration the anatomical structure of these organs and localization of the neuronal mitochondria. Cortical tissue is composed mainly of two types of cells: the highly heterogeneous population of neurons and the neuroglial cells (or glia). Glia cells outnumber the neurons by far [63–66]. Some authors suggested that the ratio of astrocyte/neuron is close to 10 [65], although this ratio may vary significantly between the brain regions. Of particular importance for the neuronal functions are macroglial cells or astroglia. Although astrocytes do not participate in the interactions between neurons on the millisecond scale, they are fundamental for supporting neurons with energy substrates and neuromediators [41, 63, 67, 68]. The importance of astroglia for the neuronal energy metabolism becomes evident from the distribution of mitochondria in the neurons.

Using a combination of immunocytochemistry, histochemistry, cytochemistry, and optical densitometry methods, Wong-Riley showed that cytochrome c oxidase (complex IV), an endogenous mitochondrial marker, correlated positively with the neuronal functional level of activity [64]. Somatic enzyme activity was found to be related to both spontaneous and synaptically evoked activities. According to the physiological effects of the cortical cells on each other, there are two categories of neurons: excitatory and inhibitory cells [63]. Excitatory cells release transmitters (mostly glutamate) at their synaptic ends that, on contact with the postsynaptic membrane, create currents that depolarize the postsynaptic cell. Inhibitory cells release transmitters (GABA) that tend to hyperpolarize the postsynaptic cell, thereby diminishing the effects of the depolarizing currents generated by the excitatory synapses [63]. Cell bodies are commonly sites of the inhibitory input, and the levels of complex IV tend to be low because repolarization subsequent to hyperpolarization is largely passive [64]. When cell bodies receive both excitatory and inhibitory synapses, as certain cortical interneurons do, the energy demand for ion pumping following depolarizing potentials increases the level of their cytochrome c oxidase activity [64].

In general, it was found that the depolarizing activities imposed a greater energy demand on postsynaptic neurons than did the hyperpolarizing activities. Therefore, dendritic metabolism makes the largest single contribution to the metabolic activity of the brain [63, 69]. As exemplified by the quantitative analysis of mitochondria in the neuropil of the primate visual cortex, 85% of mitochondria were localized in dendrites and axon terminals, whereas glial cells accounted for less than 5% of the total count of mitochondria [64].

Sokoloff et al., using the autoradiographic [^14^C]deoxyglucose method, have demonstrated that energy metabolism increased almost linearly with the degree of functional activation, that is, spike frequency, in the terminal projection zones of activated pathways [67]. The increased metabolism was found in neuropil and was minimal or undetectable in neuronal cell bodies and myelinated axons. During increased neuronal activity, increased oxygen consumption occurs locally, in the small volumes of synaptic junctions, while further propagation of the signals is supported by anaerobic glycolysis in the much larger volumes of the axons and cell body [70, 71]. This explains why energetic cost in terms of glucose of the brain functions is a linear function of the number of neurons and the estimated glucose use per neuron is remarkably constant, varying only by 40% across the six species of rodents and primates (including humans) [66].

**Figure 1:**
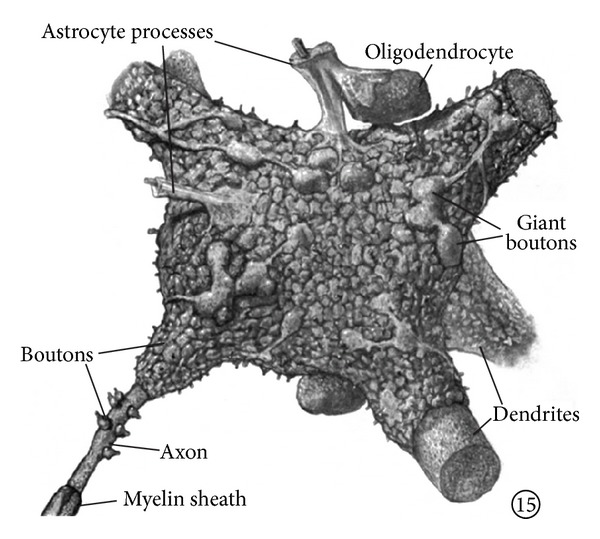
Motoneuronal perikaryon and its synaptic covering Motoneuronal perikaryon and its synaptic covering. Parent fibers are not shown. Dendrites are covered with boutons at all distances from cell body. Notice astrocytic processes cover some of oligodendrocytic surface as well as motoneuronal. (Adapted from Poritsky [72]).

In the cortex, approximately 85–90% of neurons are excitatory, and only 10–15% are inhibitory [63, 69]. Abeles [63] estimated a synaptic density of 8 × 10^8^ per 1 mm^3^ in all cortices. This density varies not much between the species. Then, in a mouse (with 100,000 neurons per 1 mm^3^), each neuron receives 8,000 synapses, whereas, in a human (with 20,000 neurons per 1 mm^3^), each neuron receives 40,000 synapses [63]. How the neuron receiving this amount of synaptic inputs might look is illustrated in Figure 1. Each neuron, depending on the size, receives synaptic inputs from hundreds or thousands of neurons.

**Figure 2:**
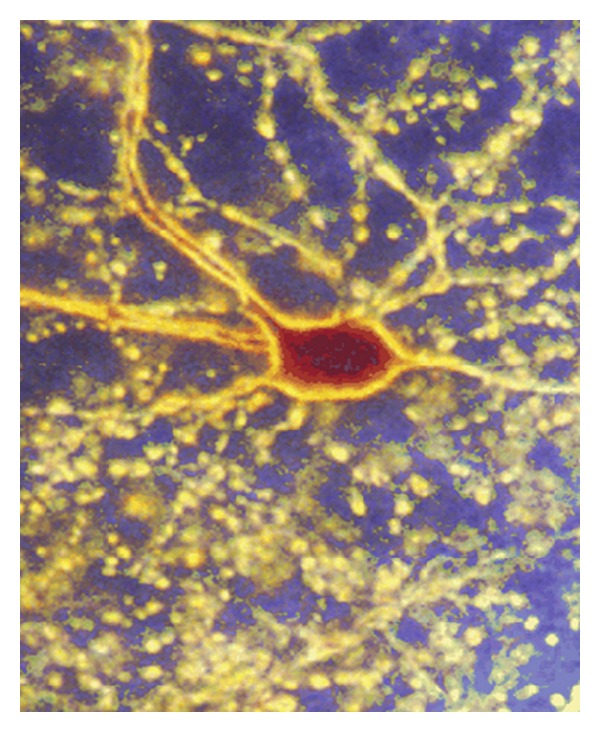
Photomicrograph of a cerebellar Golgi cell. The cell was juxtacellularly filled with neurobiotin and viewed in dark field at magnification of 240x. The soma, principal dendrites, and a diffuse cloud of axon terminals are visible, each terminal corresponding to a contact with one of thousands of granule cells (adapted from Holtzman et al. [73]).

Figure 2 illustrates the look of a relatively small cerebellar Golgi cell: one can see the cell body, principal dendrites, and a diffuse cloud of axon terminals where mitochondria are located. From these calculations and visual illustrations we can conclude that in the central nervous system most mitochondria are located in very small volumes. This excludes colocalization of any metabolic enzyme that could supply mitochondria with sufficient amount of respiratory substrates, glycolytic enzymes for example.

**Figure 3:**
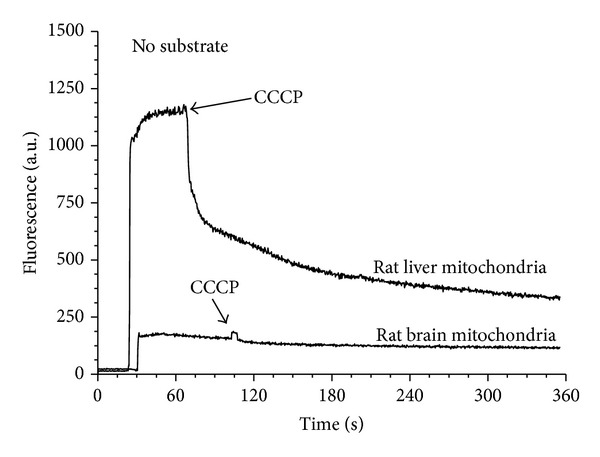
Mitochondrial pyridine nucleotides in freshly isolated rat liver and brain mitochondria incubated in the absence of added substrates. Incubation medium contained 120 mM KCl, 10 mM NaCl, 2 mM MgCl_2_, 2 mM KH_2_PO_4_, 20 mM MOPS, pH 7.2, 1 mM EGTA, and 0.7 mM CaCl_2_. Mitochondrial protein 0.5 mg/mL. Fluorescence of mitochondrial NAD(P)H was measured as described in [74].

In full accord with this suggestion, we have found that isolated nonsynaptic brain mitochondria (BM) and spinal cord mitochondria (SCM) do not contain endogenous substrates: upon addition of freshly isolated BM or SCM to the polarographic chamber without substrates, the oxygen consumption and reduction of mitochondrial NAD(P) were close to zero. This is in striking contrast to fresh liver mitochondria, which can respire for many minutes without added substrates, and, after addition of uncoupler (CCCP), it takes about 15 minutes for mitochondrial NAD(P)H to become fully oxidized (Figure 3). Freshly isolated mitochondria from the heart, kidney, and skeletal muscle also demonstrate relatively long respiration on endogenous substrates (not shown).

Estimations of energy distribution between various brain functions have shown that about 90% of energy is spent on presynaptic fluxes of Na^+^, K^+^, Cl^−^, and Ca^2+^ and less than 10% for transmitter recycling and metabotropic responses of spines [63, 69]. Thus, most of the energy is spent on synaptic activities leaving very little energy for housekeeping of neurons.

All events at synaptic junctions occur in a very short time scale (msec). Therefore, diffusion of pyruvate or lactate, produced by glycolysis, along the neuron's axons and dendrites would not be fast enough to supply substrates for mitochondria located at synaptic terminals. Under these conditions, the only energy for synaptic activities may come from synaptic and postsynaptic mitochondria oxidizing substrates provided by astroglia that embrace the synapses with its processes [75].

It should also be kept in mind that the radioactively labelled glucose and deoxyglucose methods do not allow identification of cellular elements in neuropil participating in the metabolic activation, for example, axonal terminals, dendrites, or astrocytic processes enveloping the synapses [67]. To our opinion, the results obtained with the radioactively labelled glucose and glucose metabolites, formed* in vivo* or* in vitro*, to study the brain's energy metabolism were misleading because they did not take into account the role of fatty acids oxidation both in astrocytes and probably in the neurons as well. In addition, in earlier works on the stoichiometry between glucose consumption and cycling of neurotransmitters (glutamate and GABA), it was assumed that almost all neurotransmitters released into synaptic cleft participated in the neuronal-astrocytic cycle [76, 77], and no pre- or postsynaptic uptake of GABA occurred [78].

## 7. Neuromediators Glutamate and GABA Serve as Substrates for Neuronal and Astroglial Mitochondria

Not so long ago, it was established that a significant fraction of glutamate is rapidly bound and transported postsynaptically by the glutamate transporter isoform, EAAT4, located juxtasynaptically in the membranes of spines and dendrites [79, 80]. At the climbing fiber to Purkinje cell synapses in cerebellum about 17% [80] or more than 50% [81] of synaptically released glutamate may be removed by postsynaptic transporters. Besides the cerebellum, EAAT4 transporter was found to be omnipresent throughout the fore- and midbrain [82]. Moreover, it was shown that although most of the EAAT2 protein is astroglial, in hippocampal slices around 15% is distributed in nerve terminals and axons and may be responsible for more than half of the total uptake of glutamate from synaptic clefts [77]. These data suggest that postsynaptic transport of glutamate into nerve terminals, where mitochondria are located [83], may occur in all brain regions. According to calculations of Brasnjo and Otis [80], in a single synapse EAAT4 glutamate transporters bind and transport postsynaptically about 1.3 ± 0.1 × 10^6^ glutamate molecules. In the brain, on average, 1 mm^3^ of tissue contains 1 × 10^8^ synapses [63, 84]. Because of the high density of synaptic contacts, the neuronal cells may be exposed to mediators released from hundreds of firing synapses. Thus, in a narrow space of spines and dendrites, several million glutamate molecules postsynaptically transported from synaptic boutons may create local cytosolic concentration of glutamate in a low mM range [10]. Consequently, neuronal mitochondria, particularly those located at the axonal or dendritic synaptic junctions, may temporarily metabolize, in addition to pyruvate, significant amounts of glutamate [10]. The GABAergic neurons reuptake a larger portion of the released neurotransmitter GABA compared to their glutamatergic counterparts [85]. GABA's degradation following release from the synapse takes place in both neuronal and astrocytic cells [61, 85, 86]. The GABA degradation forms succinate [61], which may stimulate oxidative stress [10].

## 8. The “Metabolic Cauldron” of Astroglia

Until recently, the functions of astroglia were considered as secondary, supporting the neuronal functions. However, more and more evidence is accumulating, which leads us to understanding that, without astroglia, the neuronal functions would, probably, never go evolutionary higher than shellfish or worms. Therefore, we have to consider functions of the central nervous system, as a result of concerted work of astroglia and neurons. The main functions of the astroglia are metabolic support of neurons with nutrients such as lactate; storing glycogen, which is the glucose reserve buffer; transport glucose and other nutrients from blood flow into the brain; neurotransmitter uptake and release and neurotransmitter synthesis* de novo*; and regulation of ion concentration in the extracellular space, as well as other functions we may not yet know. A simple enumeration of the astrocyte's functions forces us to admit that astroglial mitochondria must constantly produce large amount of energy to support these functions [43]. Taking into consideration that up to 20% of the total brain's energy expenditures are satisfied by oxidation of fatty acids [56, 87], it is clear that, without considering fatty acids as a source of energy and the carbon skeleton for the* de novo* synthesis of neuromediators, it is impossible to understand how astrocytes function.

## 9. Current Views on Energy Metabolism in Astroglia

According to estimation of Wong-Riley, astrocytes contain about 5% of the total brain mitochondria [64]. Lovatt et al. using the microarray analysis showed that astrocytes and neurons each express transcripts predicting individual self-sufficiency in both glycolysis and oxidative metabolism [75]. Importantly, most enzymes in the tricarboxylic acid (TCA) cycle were expressed at higher relative levels in astrocytes than in neurons. Mass spectrometric analysis of the TCA cycle intermediates confirmed that freshly isolated adult astrocytes maintained an active TCA cycle, whereas immunoelectron microscopy revealed that fine astrocytic processes encompassing synapses contained a higher density of mitochondria than the surrounding cells [75]. Genoud et al. have shown that synaptic release of glutamate leads to an increased astrocytic coverage of the bouton-spine interface and an increase in glutamate transporter expression in astrocytic processes [88]. These observations indicate that astrocytes exhibit a vigorous oxidative metabolism in the intact adult brain and do not rely on just glycolysis as a major source of ATP as was suggested earlier [75].

The recognition of the large scale postsynaptic transport and utilization of glutamate and GABA as mitochondrial substrates suggest that a significant amount of amino acids are removed from the pool of neurotransmitters. The key enzymes involved in the* de novo* synthesis of glutamate and glutamine, which is also a precursor of GABA in neurons, are located in astroglia [85, 89–92]. Therefore, a large portion of mitochondrial energy and substrates must be spent in astrocytes for the anaplerotic replenishment of neurotransmitters in addition to the simultaneous provision of lactate for neuronal mitochondria [28, 42, 43].

**Figure 4:**
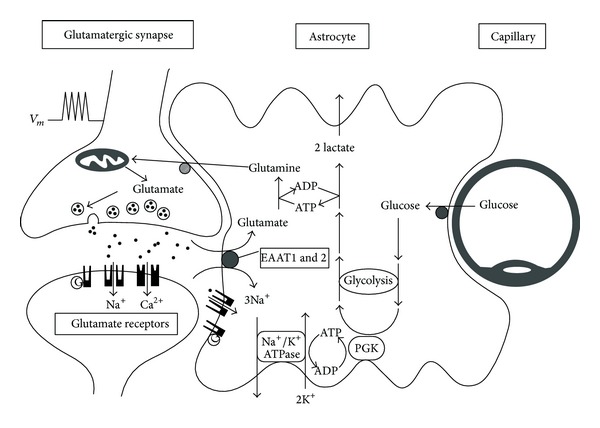
Schematic representation of the mechanism for glutamate-induced glycolysis in astrocytes during physiological activation (from [93]). At glutamatergic synapses, presynaptically released glutamate depolarizes postsynaptic neurons by acting at specific receptor subtypes. The action of glutamate is terminated by an efficient glutamate uptake system located primarily in astrocytes. Glutamate is cotransported with Na^+^, resulting in an increase in the astrocytic sodium concentration leading to activation of the astrocyte Na^+^/K^+^-ATPase. Activation of the Na^+^/K^+^-ATPase stimulates glycolysis, that is, glucose consumption and lactate production. Lactate, once released by astrocytes, can be taken up by neurons and serves them as an adequate energy substrate. (Note: from further reading of the paper, it becomes clear that “PGK” should, in fact, be “PDH,” pyruvate dehydrogenase, which provides acetyl-CoA to the TCA cycle and further electrons to respiratory chain of mitochondria.)

Neurons contribute at most 50% of cerebral cortical volume, and high astrocyte/neuron ratio is a feature of most brain regions [65]. The cytological and metabolic relationships of neurons with astrocytes and blood capillaries were well described by Magistretti and Pellerin and schematically shown in Figure 4 [93]. The legend to Figure 4 (cited from [93]) describes the essence of the original concept of the coupling mechanism between neuronal activity and glucose utilization. The concept involves activation of the aerobic glycolysis in astrocytes and lactate consumption by neurons, known as the astrocyte-neuron lactate shuttle hypothesis (ANLSH) [38, 40, 41, 93]. Glutamate uptake into astrocytes is driven by the electrochemical gradient of Na^+^; it is a Na^+^-dependent mechanism with the stoichiometry of three Na^+^ ions cotransported with one glutamate molecule [93]. Much of the glutamate taken up by astrocytes is converted to glutamine and released into the synaptic cleft for uptake by neurons to be used for resynthesis of neurotransmitters glutamate and GABA or utilized for energy [94].

In the astrocyte, glutamate is predominantly converted to glutamine by the ATP-dependent glutamine synthase, which is located almost exclusively in astrocytes [90–92, 95, 96]. Another consequence of the transporter-mediated glutamate uptake and conversion to glutamine is stimulation of aerobic glycolysis in astrocytes [38, 93]. This metabolic effect of glutamate is expressed with EC_50_ of about 80 *μ*M glutamate [97].

During neuronal activation, reuptake of glutamate by astrocyte induced a rapid (within a few seconds) and reversible increase of glucose transport from the blood into astrocytes [98] that parallels the increase in glucose utilization [31].* In vivo* activation studies in normal subjects revealed that the rise in consumption of blood-borne glucose usually exceeded the increase in oxygen consumption [28, 98]. The two processes perform on a timescale consistent with physiological processes occurring* in vivo* upon brain activation [38]. About 60% of the glucose consumed is converted in astrocytes to lactate [42]. Over several decades, dozens of studies performed* in vitro*,* ex vivo*, and* in vivo *have repeatedly demonstrated that lactate constitutes an excellent oxidative substrate for neurons (reviewed in [34, 42]). Lactate was found to be the preferable oxidative substrate over glucose in cultured neurons [47, 99].

The astrocyte-neuron lactate shuttle hypothesis was a great breakthrough in understanding how neuronal synaptic activity is supplied with substrates for mitochondrial respiration. However, Dienel in the recent comprehensive review described alternative views on the formation and metabolic fates of lactate in the brain [44]. Anyway, neither the ANLSH hypothesis [40, 41] nor alternative hypotheses [31, 44] did incorporate the fact that up to 20% of the brain's energy metabolism is supplied by the *β*-oxidation of fatty acids in astrocytic mitochondria [56, 87]. Without this, it is very difficult to explain rationally the sources of energy for anaplerotic functions of astroglia and the simultaneous existence of aerobic glycolysis, which converts large amount of glucose into lactate. In addition to the energy, we should also remember that, in some animals or human metabolic phenotypes, the glucose may be in short supply. Therefore, if *β*-oxidation of fatty acids would supply carbons for anaplerotic synthesis* de novo* of glutamate and glutamine, this would spare glucose for other functions.

It is not that the researchers did not see the dilemma of how to reconcile the simultaneous existence in the astrocyte of active aerobic glycolysis and high necessity in the oxidatively produced ATP to meet the anaplerotic functions [28, 42]. What, for example, Bouzier-Sore and Pellerin have written in their recent review about the “coexistence of what appears to be a Pasteur Effect with a Warburg Effect” [42]: “it became quite evident that astrocytes appear to be very versatile cells in terms of metabolism. Although they have an important oxidative metabolism especially toward glutamate as described above, they also exhibit a clear aerobic glycolysis capacity… Indeed, raising oxygen levels promote oxidative metabolism in astrocytes at the expense of anaerobic glycolysis and lactate production (Pasteur Effect). But even in presence of supraphysiological levels of oxygen (e.g., 21% O_2_ in culture conditions), aerobic glycolysis with lactate production was shown to take place in astrocytes (Warburg Effect), which can be further enhanced under certain circumstances (e.g., glutamate exposure). The capacity to exhibit both processes may depend on the expression of particular subsets of proteins that need to be specifically identified.”

## 10. *β*-Oxidation of Fatty Acids by Astrocytic Mitochondria as a Source of Energy and Carbons for Anaplerotic Functions

We have already mentioned that the astrocytic perisynaptic processes contain a higher density of mitochondria than surrounding cells [75]. This means that upon neuronal activation, astrocytes simultaneously supply neurons with lactate as a substrate for neuronal mitochondria, whereas astrocytic mitochondria supply high levels of ATP to maintain the glutamate/glutamine cycle and other ATP-consuming functions. We believe that this apparent discrepancy can be solved, if we introduce *β*-oxidation of fatty acids into the astrocytic metabolic caldron.

**Figure 5:**
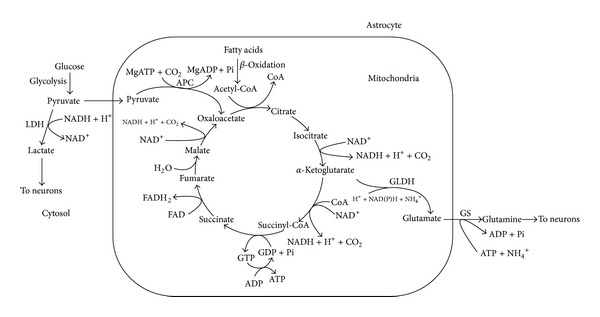
Simultaneous operation of aerobic glycolysis and the fatty acids supported tricarboxylic acid cycle and oxidative phosphorylation in the astrocyte. Enzymes: LDH: lactate dehydrogenase, APC: ATP-dependent pyruvate carboxylase, 1–9: the enzymes of the TCA cycle, which operates clockwise, GLDH: glutamate dehydrogenase, and GS: glutamine synthetase.

Figure 5 illustrates our first attempt to reconcile the oxidative metabolism of fatty acids and the aerobic glycolysis in astrocytes.

The current paradigm suggests that astrocytes utilize glucose both for aerobic glycolysis and for oxidative phosphorylation. The selection of the metabolic pathway for glucose occurs at the level of pyruvate. To direct pyruvate to the respiratory chain, the mitochondrial pyruvate dehydrogenase complex (PDHC) must convert pyruvate to acetyl-CoA in the overall reaction: pyruvate + NAD^+^+ CoA → acetyl-CoA + NADH + CO_2_. Acetyl-CoA after condensation with oxaloacetate (OAA), catalyzed by the citrate synthase, enters the tricarboxylic acid (TCA) cycle as citric acid. Part of PDHC is located in the mitochondrial matrix, but most of the enzyme is associated with the inner membrane (100) where it may form functional complexes with other enzymes of the TCA cycle. According to our hypothesis, presented here (see Figure 5), *β*-oxidation of fatty acids is the source of acetyl-CoA for the TCA cycle. Oxaloacetate for the citrate synthase reaction is provided by the pyruvate carboxylase, which is exclusively the astrocytic enzyme [100–102]. Waagepetersen et al. concluded that neuronal pyruvate carboxylation is unlikely to be of quantitative significance [102]. Pyruvate carboxylase catalyzes the ATP-dependent irreversible carboxylation of pyruvate to form oxaloacetate: pyruvate + HCO_3_^−^ + ATP → oxaloacetate + ADP + Pi.

The idea that *β*-oxidation of fatty acids may be the preferable substrate for production of acetyl-CoA in astrocytes is supported by the properties of the PDHC in astrocytic mitochondria. Halim et al. have shown that all components of the PDHC were expressed in both neurons and astrocytes in culture [103]. However, in astrocytes, the PDHC activity was kept strongly inhibited through phosphorylation of the pyruvate dehydrogenase alpha subunit. In contrast, neuronal PDHC operated close to maximum with much lower levels of phosphorylated PDH alpha. Dephosphorylation of astrocytic PDH alpha restored the PDHC activity and lowered lactate production [103]. This intrinsic property of the astrocytic mitochondrial PDHC will favor glucose entering the glycolytic pathway and production of OAA, rather than entering the TCA cycle. This also suggests that, in astrocytes, fatty acids may be the major source for acetyl-CoA.

Upon neuronal activation, aspartate increased glutamate synthesis in both control and the aralar-deficient astrocytes, mainly by serving as amino donor [96], but also by producing additional OAA and thus saving more glucose for lactate production. Under these conditions, *α*-ketoglutarate will be constantly produced in the TCA cycle and converted by the mitochondrial aspartate aminotransferase (AAT) to glutamate [104]. It has been shown that mitochondrial glutamate dehydrogenase (GLDH) is important for glutamate degradation, whereas glutamate biosynthesis occurs mainly as a transamination via AAT [104]. It is important that both in neurons and astrocytes AAT and GDLH may function as multienzyme complexes [104]. The complex regulation of glutamate formation or disposal by the multienzyme complexes is discussed in the comprehensive review by McKenna et al. [104].

The newly produced glutamate will be actively removed from mitochondria by the cytosolic ATP-dependent glutamine synthase, and at high cytosolic ATP, the phosphorylated glutamine transporters will pump out glutamine from the astrocyte [105].

## 11. In Astrocytes, Oxidative Phosphorylation Proceeds Simultaneously with Aerobic Glycolysis

Upon neuronal activation, astrocytes remove significant part of glutamate from the synaptic cleft by the Na^+^-dependent uptake system, which requires energy, and thus stimulates glycolysis [31, 38]. Glutamate induces also a rapid (within a few seconds) and reversible increase of glucose transport into astrocytes [106] that parallels the increase in glucose utilization [31, 38]. Moreover, glutamate may cause inhibition of neuronal glucose transport, which is even stronger in the presence of lactate [107]. The primary isoforms of glucose transporter in brain are GLUT1, detected at high concentrations in blood-brain barrier and glia; GLUT3 in neurons; and GLUT5 in microglia [108]. After glucose entry, glucose is phosphorylated by type I hexokinase (HK1) [109, 110]. In astrocytes, most of HK1 is associated with mitochondria and the activity of HK1 bound to mitochondria is greater than the cytosolic hexokinase [111, 112]. Moreover, this association of HK1 is modulated in coordination with changes in the cell's metabolic state [111]. Thus, in the astrocytes, formation of glucose-6-phosphate occurs not for the expense of the cytosolic ATP but for the expense of mitochondrial ATP and this will stimulate formation of pyruvate.

Neurons lack pyruvate carboxylase; instead, all activities of this enzyme were found in astroglia [113, 114]. In addition, PDHC in astrocytic mitochondria is inhibited [75], very similar to liver mitochondria when the liver metabolism switches from glucose oxidation to gluconeogenesis and oxidation of fatty acids [115]. Together, these properties of the key enzymes in astrocytes direct more pyruvate to formation of lactate and OAA. This will also promote *β*-oxidation of fatty acids that will supply the TCA cycle with acetyl-CoA, which is allosteric activator of pyruvate carboxylase [116]. Upon neuronal activation, OAA produced by pyruvate carboxylase will be directed toward the TCA cycle and *α*-ketoglutarate will be removed from the cycle for glutamate synthesis; in resting synapses, when the necessity in newly formed glutamate and lactate is diminished, the OAA will be directed towards gluconeogenesis, which also requires ATP. Again, *β*-oxidation of fatty acids will provide ATP for this energy-dependent metabolic pathway and more glucose will be stored as glycogen.

Active oxidative phosphorylation will maintain in the cytosol high ATP/ADP and NADH/NAD^+^ ratios. This is because *β*-oxidation enzymes, the membrane-bound TCA cycle enzymes, and the respiratory chain may be organized in the functional complexes and work relatively independently from the cytosolic pool of ATP and NADH. Therefore, a large part of pyruvate formed during glycolysis will be reduced to lactate, which will be rapidly removed from the astrocytes [38]. In this way, both the aerobic glycolysis and oxidative phosphorylation will proceed simultaneously in the astrocyte as quasi-irreversible pathways, which were metabolically activated by transported glutamate from the activated synapses.

When the neuronal synaptic terminals become “quiet,” astrocytes will also become metabolically inactivated because accumulation of lactate will inhibit glycolysis, *α*-ketoglutarate will not be converted to glutamate and thus will be metabolized in the TCA cycle towards OAA. Altogether, this will stimulate gluconeogenesis and accumulation of glycogen. Glycogen is the glucose reserve buffer during periods of high rate of glucose consumption and glucose shortage. To our opinion, glucose from the glycogen during periods of activation will not be released to neurons but, rather, will be converted to lactate inside the astrocyte. Thus oxidation of fatty acids by astroglial mitochondria provides energy and part of the carbon skeleton for the anaplerotic synthesis of neuromediators and saves glucose, which is converted either into lactate to fuel neurons, or stored for emergency as glycogen.

## 12. Properties of Neuronal Mitochondria

In experiments with isolated forebrain or spinal cord mitochondria, we, as well as many other researchers, used the popular method by Sims [117] with slight modifications [10, 11]. The method uses a very mild homogenization technique and a purification step with the discontinuous Percoll gradient. The resulting mitochondria are considered to originate predominantly from postsynaptic elements of the synapses, partly from the blown presynaptic vesicles, neuronal cell bodies, and, possibly, astroglia [45, 84]. The “contamination” with the astroglial mitochondria is, more likely, negligible because they showed high rates of respiration with glutamate and pyruvate. As we have mentioned above, astrocytic mitochondria have low expression of glutamate-aspartate transporter [46, 118] and low activity of PDHC [103]. Electron microscopic study has shown that, purified in the Percoll gradient, brain and spinal cord mitochondria were not contaminated by other organelles [16]. Panov et al. [10] have shown that nonsynaptic mitochondria have respiratory qualities similar to those found for synaptic mitochondria [60]. In this review, we will designate these, the so called nonsynaptic mitochondria, as brain mitochondria (BM).

## 13. Brain but Not Astroglial Mitochondria Possesses the Electrogenic Glutamate/Aspartate Transporter (GAT)

Brain mitochondria have some principal properties that strongly distinguish them from the astrocytic mitochondria. First, astroglial mitochondria have low or no expression of the glutamate/aspartate transporter (GAT), but, instead, GAT is mainly neuronal [45, 118–120]. The glutamate-aspartate transporter is important metabolically because it is a required component of the malate/aspartate shuttle (MAS) [120, 121]. The malate-aspartate shuttle is considered the most important shuttle in brain. It is particularly important in neurons and may be extremely low or even nonexistent in brain astrocytes [59, 120]. Hertz suggested that aralar/AGC1 in brain astrocytes, even at very low levels, could also play a role in a modified aspartate-malate shuttle to oxidize reducing equivalents in mitochondria [59]. MAS is the major pathway by which cytosolic electrons from NADH can enter the mitochondria and be oxidized [120, 121]. Lactate, as a substrate, is energetically richer than pyruvate, but without MAS; neuronal mitochondria cannot effectively utilize this extra hydrogen and convert lactate to pyruvate.

The absence of GAT from astrocytes in the brain implies a compartmentation of the intermediary metabolism of glucose, with glycolysis taking place in astrocytes and lactate exported to the extracellular fluid and oxidized to CO_2_ and H_2_O in neurons [43]. Glycolysis starting from glucose can, of course, proceed actively in neurons as well as in astrocytes, but the very fact that most neuronal mitochondria are localized in the narrow space of synapses, suggests that glycolysis cannot be the major source of mitochondrial substrates for synaptic activities. However, propagation of the electrical signal, along dendrites, axons, and cell body, is maintained with participation of the glycolytic ATP.

GAT is expressed in mammals in two isoforms, aralar and citrin [122]. Aralar is expressed in the liver and both isoforms are expressed in the heart. Although both isoforms are detected in the brain during the first weeks of life, only aralar is detected in the mature brain [118]. Both aralar and citrin are electrogenic and unidirectional. They transport a protonated glutamate into the mitochondria in exchange for aspartate anion utilizing the energy of the mitochondrial transmembrane electrochemical gradient (ΔΨ) [121].

Berkich et al. stressed that aralar requires the presence of mitochondrial aspartate aminotransferase (AST) to generate aspartate and cannot provide glutamate for glutamate dehydrogenase (GLDH) because GLDH does not produce aspartate for exchange with glutamate [45]. However, both neurons and astroglial cells have two glutamate/hydroxyl carriers, GC1 and GC2 [122, 123]. These carriers operate reversibly providing substrate for GLDH.

In the energized cells, active transport of glutamate into neuronal mitochondria by the electrogenic aralar, which is structurally and functionally coupled to AAT, will produce the reciprocal amount of aspartate. Aspartate may be transported into astrocytic cells, where it will be converted by the cytosolic AAT into glutamate and oxaloacetate (OAA) [68].

## 14. Isolated Brain Mitochondria Have Strongly Inhibited Succinate Dehydrogenase

**Figure 7:**
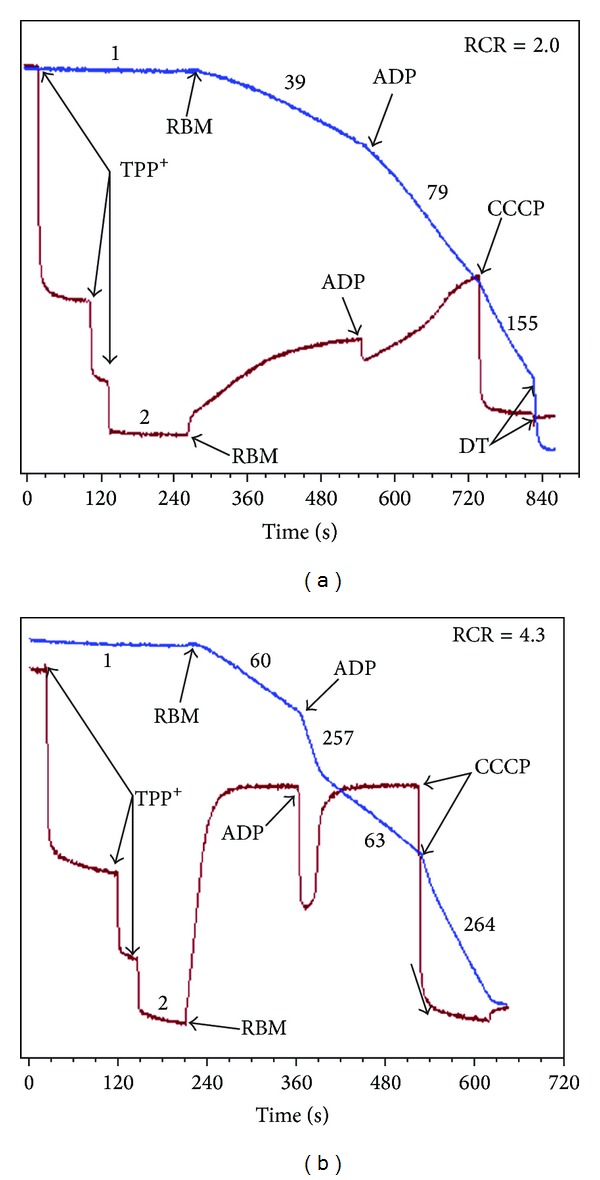
Respiratory activity and membrane potential of the rat (Lewis) brain mitochondria isolated in the absence (a) and in the presence (b) of 0.1% BSA. Incubation conditions: 125 mM KCl, 10 mM NaCl, 10 mM MOPS, pH 7.2, 2 mM MgCl_2_, 2 mM KH_2_PO_4_, 1mM EGTA, and 0.7 mM CaCl_2_. At Ca^2+^/EGTA = 0.7, the [Ca^2+^]_Free_ was 1 µM. Chamber volume = 0.65 mL. Substrate: succinate 5 mM, no rotenone was added. Additions: BM 0.3 mg, ADP 150 *μ*M, and CCCP 0.5 *μ*M. Numbers at the traces are respiratory activities in nmol/min/mg mitochondrial protein. Respiratory activity ratio (RCR) is *V*_State 3_/*V*_State 4_.

A large scale utilization of glutamate by BM, as the energy-delivering substrate, is strongly suggested not only by the documented large scale transport of the mediator into neurons [80, 124] but also by the properties of isolated BM and SCM [9, 10, 16, 62, 125]. In a number of recent works, we have shown that in the brain and spinal cord mitochondria, the major source of reactive oxygen species (ROS) production was associated with the reverse electron transport (RET) [10, 125]. Therefore, one of the most interesting and important features of the neuronal mitochondria was that mitochondrial succinate dehydrogenase (SDH) was usually strongly inhibited (see Figure 7) by endogenous oxaloacetate (OAA) [10, 105]. The degree of this intrinsic SDH inhibition varies strongly between the species. In other words, it depends on the metabolic phenotype of the BM [62, 126].

The significance of this inhibition of SDH becomes evident from the fact that most neuronal mitochondria are located in the narrow spaces of synaptic junctions [63, 64]. Upon activation, the synaptic mitochondria have no other function except restoration of the ionic homeostasis across the membranes of the synapses. Therefore, when synapses are not activated, the inactive mitochondria become hyperpolarized. In other words, the synaptic mitochondria will respire in the metabolic State 4, when the reverse electron transport (RET) activates production of reactive oxygen species (ROS) on complex I [9, 10]. This is in strict contrast to mitochondria in other organs, such as liver, kidney, and heart, where mitochondria are constantly producing ATP and the RET is usually at minimum.

Brain (BM) and spinal cord (SCM) mitochondria isolated in the absence of bovine serum albumin (non-BSA-BM) usually display strong inhibitions of succinate oxidation (see Figure 7(a)). Therefore, most researchers traditionally isolate BM and SCM in the presence of defatted BSA [126], which significantly improved oxidation of succinate (Figure 7(b)). Figure 7(b) shows, however, that even with the BSA-BM oxidizing succinate, it required more than a minute for the membrane potential to reach the maximum.

**Figure 8:**
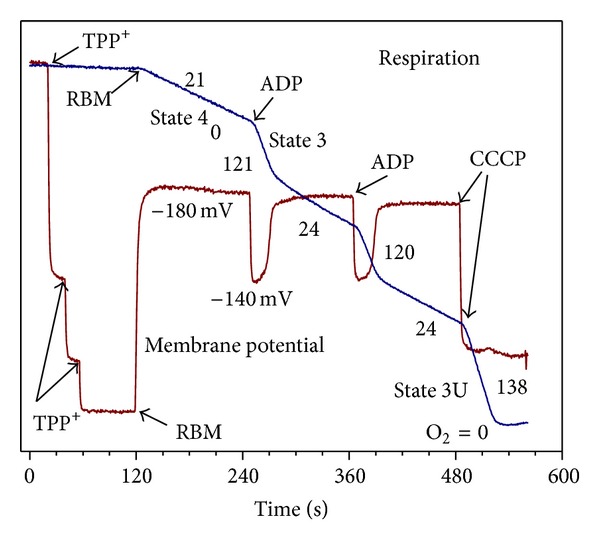
Respiratory activity and membrane potential of the rat (Sprague Dawley) brain mitochondria isolated in the absence of 0.1% BSA. Incubation conditions as in Figure 7. Substrates: pyruvate 2.5 mM, malate 2 mM. Additions: ADP 150 *μ*M and CCCP 0.5 *μ*M. Numbers at the traces are respiratory activities in nmol/min/mg mitochondrial protein.

It should be noted that, with many species, the BM isolated in the presence of BSA and initially oxidizing succinate develop strong inhibition of SDH after 40–60 minutes after isolation. For comparison, in the non-BSA-BM oxidizing pyruvate + malate (Figure 8), energization of BM, as measured by a TPP^+^-sensitive electrode, reached the maximum within few seconds.

It must be mentioned that too many researchers used succinate as a substrate in the presence of rotenone, which prevented formation of OAA, but also abolished other physiologically important events, such as reverse electron transport and the associated ROS production. We believe that intrinsic inhibition of SDH is an evolutionary mechanism against oxidative damage of the most vulnerable synaptic mitochondria. This is of particular importance because we have shown that in the BM and SCM up to 50% of pyruvate and glutamate are oxidized via alanine and aspartate aminotransferases, which produce *α*-ketoglutarate and then succinate [10, 16]. Another adaptive mechanism in BM and SCM against excessive production of ROS in the synaptic mitochondria of quiescent neurons is probably the low contents of the intramitochondrial substrates.

## 15. Metabolic Phenotypes of Mitochondria due to Different Affinities of SDH to Oxaloacetate

The intrinsic inhibition of succinate oxidation in BM and SCM varies significantly between the species [62, 126]. SCM in general showed much lower inhibition of SDH as compared to the BM together with the lower rates of respiration with all substrates. This resulted in the lower rates of ATP provision and increased rate of the RET-dependent ROS production in resting SCM [16, 62, 125]. BM and SCM have very high activities of the matrix superoxide dismutase (SOD2) that very rapidly converts superoxide radicals to H_2_O_2_ [10, 62]. Therefore, it is very likely that most of the damaging effects of increased ROS production are associated with the increased H_2_O_2_ [62, 127]. We have recently provided evidence that the haplotype-specific differences in production of ROS (H_2_O_2_) may be responsible for development of sporadic and familial cases of amyotrophic lateral sclerosis (ALS) [62]. Evidently, the degree of the intrinsic inhibition of SDH may be important phenotypic feature that determines the susceptibility of a particular organism to diseases, such as ALS, Alzheimer's disease, and Parkinson's disease, where the increased oxidative stress plays important pathogenic roles.

**Figure 9:**
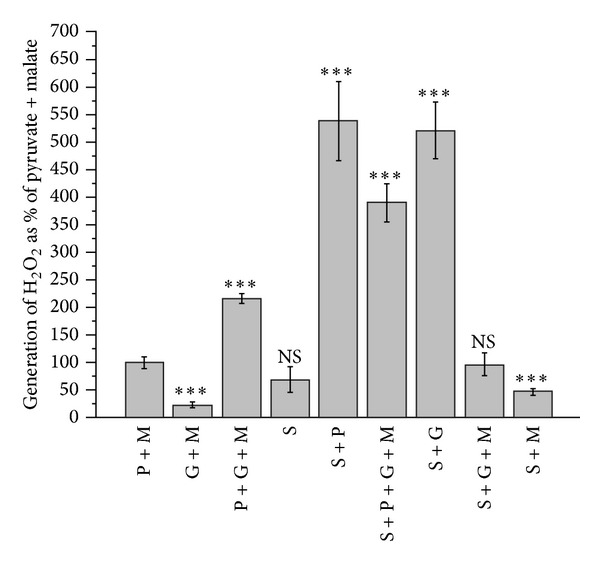
Generation of H_2_O_2_ by non-BSA rat brain mitochondria oxidizing various substrates and substrate mixtures. Incubation conditions as in Figure 7. Substrates: pyruvate 2.5 mM, glutamate 5 mM, succinate 5 mM, and malate 2 mM. The method of ROS measurements was described in [10]. Statistics: ***P < 0.001; NS: not significant.

There may be several mechanisms responsible for variations in the OAA-dependent intrinsic inhibition of SDH. OAA is the most powerful inhibitor of SDH [128]. The affinity of SDH for OAA changes with reduction of the enzyme's sulfhydryl group depending on mitochondrial energization. Upon deenergization of mitochondria, the affinity of SDH to OAA may increase tenfold [129]. Another important property of SDH is that, besides succinate, the enzyme can also oxidize malate with the similar affinity [130]. While externally added OAA competes with succinate for binding to the enzyme, OAA formed during oxidation of malate remains tightly bound to the enzyme, which makes malate a powerful inhibitor of SDH [130]. Of interest, the metabolic phenotype (nontransgene) of the Sprague Dawley rats (from Taconic Farms Inc. Germantown, NY 12526) in 2007 displayed a strong inhibition of ROS production in the presence of malate (Figure 9).

This ability of malate to inhibit ROS production was lost in the nontransgene Sprague Dawley rats in 2010, when the animals failed to develop ALS symptoms upon receiving the mutated human SOD1 gene [62]. In this metabolic phenotype, the presence of malate in the substrate mixtures stimulated production of the RET-dependent ROS (data not shown).

## 16. Activation of Oxidative Phosphorylation during Simultaneous Oxidation of Pyruvate, Glutamate, and Malate

In BM, there are close functional and structural relationships between enzymes that metabolize the tricarboxylic acid (TCA) cycle intermediates and amino acids [131]. There are three enzymes, ALT, AAT, and GLDH, which, in BM, convert alanine and glutamate to *α*-ketoglutarate (*α*-KG) [94, 132]. The enzymes are found structurally colocalized and may form functional complexes without releasing the intermediary metabolites [68, 94]. This type of enzymes interaction is important for the neuronal mitochondria, which have a very high content of proteins in the matrix. For this reason, the matrix of mitochondria from the heart, brain, and skeletal muscle is a hard gel, which excludes or greatly hampers the diffusion of small molecules [133, 134]. Therefore, many enzymes of the TCA cycle are tightly associated with the inner mitochondrial membrane. For example, the membrane-bound pools of pyruvate dehydrogenase complex (PDHC) and *α*-ketoglutarate dehydrogenase complex (KGDHC) about 200 times exceed the “soluble” pool of these enzymes in the matrix [133]. The enzymes are organized into stable functional complexes, which tunnel metabolites along the complexes forming efficient metabolic channels. Such multienzyme complexes have been shown for *β*-oxidation of fatty acids [135], malate-aspartate shuttle (aralar) with AAT and the TCA cycle enzymes [136, 137], AAT and GLDH [68], and respiratory chain complexes [138, 139]. It was shown that, for the heart mitochondria, the ratio for oxidative phosphorylation (OXPHOS) complexes I : II : III : IV : V was 1 : 2 : 3 : 6-7 : 3–5 [140] or more recently determined as 1 : 1.5 : 3 : 6 : 3 [141]. The respiratory complexes interact with each other to form a supercomplex named the respirasome [138]. Based on the above ratios of the OXPHOS complexes, Schägger and Pfeiffer [139] suggested that the respirasome exists as a mixture of two large supercomplexes and one smaller supercomplex. Each of the two large supercomplexes is comprised of a complex I monomer, a complex III dimer, and four copies of complex IV. The smaller supercomplex contains two complexes III and four complexes IV [16, 118]. The major advantages of the supercomplex structure of the mitochondrial respiratory chain are substrate channeling, catalytic enhancement, sequestration of reactive intermediates, and structural stabilization [138, 139]. Evidently, similar considerations consider the neuronal and astroglial mitochondria, which also have high respiratory activities and complex organization of the metabolic enzymes.

We suggest that, in addition to the benefits listed above, the respirasome is also an evolutionary adaptive mechanism designed to prevent excessive production of ROS. Several-fold excess of complex IV in the clusters of respiratory enzymes promotes oxidation of the potential sites of superoxide radical production in complexes I and III [9]. Evidently, this mechanism was developed early during the evolution of the aerobic organisms because it is available in aerobic bacteria and yeast [138, 139]. In addition, mitochondria* in vivo* oxidize several different substrates simultaneously coming from different metabolic pathways, which are strongly organ and species specific [10, 16], and thus may have different impacts on oxidative stress.

The three glutamate-transforming enzymes have high Michaelis constants for glutamate: for GLDH 8–17 mM [142] and for ALT and AST in the range of 7–20 mM [132]. However, in view of the electrogenic nature of the aralar and removal of *α*-KG by the TCA cycle [45], in energized BM, the three glutamate-transforming reactions operate irreversibly with the net loss of glutamate. Therefore, even at concentrations much lower than the Km's of the enzymes, glutamate will be rapidly oxidized.

During neuronal activation, astrocytes provide additional amounts of lactate for postsynaptic neuronal mitochondria [34, 143, 144]. For rapid conversion of lactate to pyruvate postsynaptic mitochondria must receive a certain amount of glutamate to fuel MAS in order to recycle the cytosolic NAD^+^ [10, 45]. Importantly, several research groups [145–148] have shown that upon neuronal activation, nanomolar concentrations of external Ca^2+^ specifically activate oxidative phosphorylation via the aralar-dependent transport of glutamate into mitochondria. Therefore, increased neuronal activity through small changes in the extramitochondrial Ca^2+^ activates MAS and OXPHOS, whereas increased mitochondrial Ca^2+^ may further activate mitochondrial respiration via specific dehydrogenases [149]. As a result, the glutamate, which is present in the neurons, may be rapidly oxidized both presynaptically and postsynaptically [10, 60].

We have suggested [10] that in the presence of pyruvate + glutamate, particularly when malate was also present (with the exception shown above), the respiration overcomes some limiting steps in the respiratory chain. We considered at least two such limiting steps [10]. First, the activity of succinate dehydrogenase (SDH/Complex II) may be inhibited by OAA and thus limit the rate of the TCA cycle operation during the state 3 and state 3U. The ability of pyruvate and glutamate to overcome inhibition of SDH was associated with the metabolic removal of OAA in the citrate synthase, aspartate, and alanine transaminase reactions, respectively.

The second limitation point is the *α*-KGDHC reaction. It has been shown [150] that activity of *α*-KGDHC is the lowest among the TCA cycle enzymes and is controlled by the availability of *α*-KG [151] and the enzyme's affinity for *α*-KG, which is controlled by Ca^2+^ and Mg^2+^ [152, 153]. In addition, a decreased matrix ATP/ADP ratio due to increased energy consumption in activated neurons would also increase the availability of GDP for the substrate level phosphorylation and thus the overall activity of *α*-KGDHC [94]. The key role of *α*-KG for the TCA-related hydrogen supply was shown in experiments with labelled metabolites [154]. Balazs (1965) has shown that the amount of *α*-KG increased 30-fold in BM oxidizing glutamate + pyruvate + malate [154]. Under these conditions, GLDH does not participate in production of *α*-KG [155]. Since both AAT and ALT are present in a great excess, as compared with the respiration rate, the OAA is continuously removed by the transamination reactions. Balazs concluded that a competition takes place between the *α*-KGDHC and GLDH, probably for NAD^+^, resulting in preferential oxidation of *α*-ketoglutarate [155].

**Figure 10:**
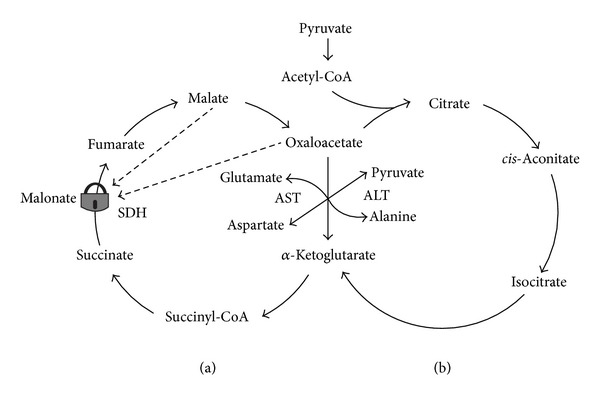
A schematic presentation of operation of the tricarboxylic acid cycle in brain mitochondria oxidizing glutamate and pyruvate. The figure was adapted from [95]. Abbreviations: AST: aspartate aminotransferase, ALT: alanine aminotransferase, and SDH: succinate dehydrogenase. The symbol of the closed lock means the step catalyzed by SDH is inhibited. The dashed arrows indicate inhibitory influences of malate and oxaloacetate on SDH.

Yudkoff et al. suggested that in the presence of glutamate + pyruvate, the TCA cycle in brain mitochondria operates as two coupled cycles (see Figure 10): cycle A is leading from *α*-KG to OAA and cycle B from OAA to *α*-KG which includes the citrate synthase reaction (see Figure 10) [60]. According to Yudkoff et al., the flux of substrates through cycle A is 3–5-fold faster than through the cycle B [60]. Thus, with pyruvate + glutamate + malate, activation of *α*-KGDHC and SDH may significantly increase the rates of the TCA cycle and respiratory chain in state 3 and state 3U (see Figure 9). A high turnover of cycle A, with activated SDH, would increase RET and the associated ROS production in resting mitochondria. As a result, the rate of oxygen consumption in the metabolic State 4 also increases [10].

## 17. Neuronal Mitochondria Do Oxidize Fatty Acids in the Mixtures with Other Substrates

It has long been recognized that fatty acids can enter the brain and be metabolized to CO_2_ and H_2_O [51, 52, 156, 157]. Much of the evidence was obtained in the* in vitro* studies using primary cultures of various cells from the developing brain [67, 156] and experiments with brain perfusion [53, 54]. It was concluded that the brain's capacity to oxidize fatty acids and the levels of the enzymes of fatty acid oxidation in the brain were much higher in the suckling rat than in the adult rat [55, 156]. In primary cultures, only astrocytes were able to utilize fatty acids for ^14^CO_2_ production, and the rate of utilization was greater than that of the ketone bodies. The metabolic patterns of the brain cells derived from the developing brain complemented the nature of the diet of the suckling animals, which was rich in fat and low in carbohydrate [156]. And there was evidence that the brain of adult animals (at least from dogs and cats) did not utilize free fatty acids* in vivo* [53, 54]. Thus, currently it is widely accepted that neuronal mitochondria in adult brain do not oxidize fatty acids [158].

**Figure 11:**
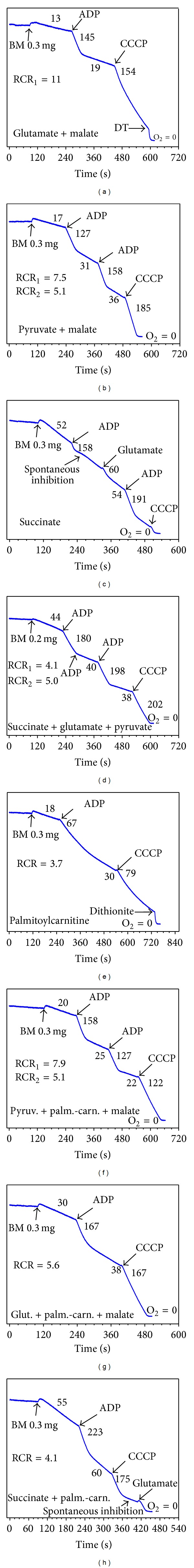
Oxygen consumption by rat (Sprague Dawley, 2010) brain mitochondria isolated without BSA oxidizing various substrates and their mixtures in different metabolic states. Experimental conditions as described in Figure 6. Metabolic states are shown in Figure 7. Substrates: glutamate 5 mM, malate 2 mM, pyruvate 2.5 mM, succinate 5 mM, and palmitoyl carnitine 25 *μ*M. Numbers at the traces are respiratory activities in nmol/min/mg mitochondrial protein. Respiratory activity ratio (RCR) is *V*_State 3_/preceeding *V*_State 4_. Additions: brain mitochondria 0.3 mg, ADP 150 *μ*M, CCCP 0.5 *μ*M, and glutamate 5 mM.

**Figure 12:**
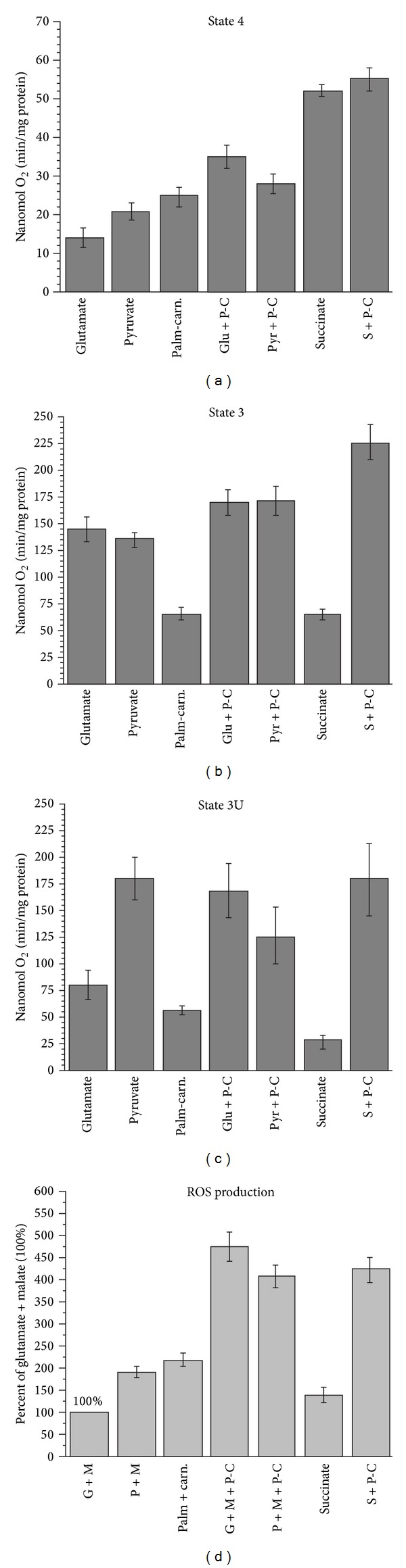
Respiratory activity and ROS production by rat brain mitochondria oxidizing palmitoyl carnitine in various substrate mixtures. Incubation conditions as in Figure 7 and substrate mixtures as described in Figure 11. The data are average of three different isolations (M ± standard error). ROS production was measured with the Amplex red method as described in [10]. The minimal rate of ROS production was observed with glutamate + malate, which was taken as 100%.

There is very little evidence regarding the fatty acids metabolism in the isolated brain mitochondria from adult animals with the use of the new paradigm, that is, with the use of the physiologically relevant substrate mixtures. We addressed this issue using rat brain mitochondria and our new methodology based on the assumption that* in situ* mitochondria oxidize not a single but several substrates. Therefore, we tested palmitoyl carnitine as a substrate in combination with the “classical” substrates for the brain mitochondria.  Figure 11 presents direct polarographic recordings of respiratory activities of the rat brain mitochondria with different substrates and their mixtures.  Figure 12 presents the summary of three separate isolations and also shows the rates of ROS production with each substrate and a substrate mixture.

The panels (a), (b), and (c) of Figure 11 illustrate respiratory activities of BM with the “classical” substrates: glutamate + malate, pyruvate + malate and succinate. Notice that with succinate (Figure 11(c)), the State 4 respiration was several-fold higher than with glutamate (Figure 11(a)) or pyruvate (Figure 11(b)). The rates of the State 4 respiration to a large degree are determined by the reverse electron transport (RET) and the associated production of superoxide radical. RET is an energy-dependent function and, since formation of the superoxide radical, serves as a sink for electrons; mitochondria increase the State 4 respiration. Thus, the State 4 respiration may serve as a qualitative indicator of changes in ROS production by energized mitochondria.

Figure 11(c) shows that due to intrinsic inhibition of SDH, the response of BM oxidizing succinate to addition of ADP was negligible. The inhibition was released upon addition of 5 mM glutamate (Figure 11(c)).  Figure 11(d) shows that, during oxidation of the substrates mixture succinate + glutamate + pyruvate, the rate of oxidative phosphorylation was significantly higher than with glutamate or pyruvate (see also Figure 12).

When palmitoyl carnitine was used as a substrate (Figure 11(e)), the BM responded to ADP by a 3-fold increase in respiration, which was followed by significant inhibition of oxygen consumption. Addition of uncoupler (CCCP) also failed to significantly increase respiration. This experiment indicates that BM are capable, albeit not efficiently, of oxidizing fatty acids. We found that addition of malate to palmitoyl carnitine had no effect on the rates of respiration. Only *L*-palmitoyl carnitine must be used as a substrate because *D*, *L*-palmitoyl carnitine is inhibitory.

In situations when palmitoyl carnitine was oxidized by the BM in the presence of either pyruvate (Figure 11(f)), glutamate (Figure 11(g)), or succinate (Figure 11(h)), the rates of respiration were significantly increased in all metabolic states (see also Figure 12). The stimulation of respiration was particularly large during simultaneous oxidation of succinate + palmitoyl carnitine (see Figure 11(h) and Figure 12).

At this time, we have no explanations on the mechanism or mechanisms that were involved in the stimulatory effect of respiration with the mixtures of palmitoyl carnitine with other substrates. This issue requires further investigation. However, the results presented in Figures 11 and 12 unequivocally demonstrate the ability of BM to efficiently utilize fatty acids as a source of energy not only in astrocytes but in the neurons as well.

Of particular importance was the fact that simultaneous oxidation of palmitoyl carnitine with the “classical” substrates resulted in the increased State 3 respiration and several-fold increase in the generation of ROS in BM (see Figure 12). This may have implications for understanding the pathogeneses of neurodegenerative diseases and pathologies associated with aging and the metabolic syndrome.

## 18. Conclusions

During the last decade, a large body of evidence has been accumulated on participation of mitochondrial dysfunctions in pathogeneses of practically all major pathologies in human [1, 8, 159, 160]. The roles of mitochondrial dysfunctions were particularly evident in a number of neurodegenerative diseases (NDD): Parkinson's disease, Alzheimer's disease, and amyotrophic lateral sclerosis (ALS) [159, 160]. Different genetic and toxic animal models have been developed to study the roles of mitochondria more closely [10, 161–163]. There is one important feature common for the NDDs and animal models of these diseases: most cases of NDDs are sporadic with only few percentage of cases having clear genetic cause [62]; regarding the animal models, for example, the rotenone model of Parkinson's disease or transgenic model of ALS, can be reproduced on only one or two species, while other species, rats in particular, did not reproduce the symptoms of a pathology [164].

According to Wallace, variations in the haplotypes of mtDNA may determine to a large degree the differences in susceptibility of human individuals and experimental animal species to a particular pathology [1, 4]. At the level of physiology, variations in the haplotypes of mtDNA may come into view as variations in the mitochondrial metabolic phenotypes. That is, mitochondria may have differences in affinity to various substrates, allosteric regulators, and so forth. These variations would be further manifested as different rates of ATP synthesis or ATP/ADP ratios and different rates of ROS production. Therefore, in order to study more closely the relationships between variant haplogroups of mtDNA and pathologies, we have to study variances in mitochondrial metabolic phenotypes. The old methodology to study mitochondrial functions using only one substrate, succinate + rotenone, in particular, is outdated. We now have several examples of advantages by using substrate mixtures in studying mitochondrial functions [10, 165]. In this review, we show that isolated brain mitochondria do oxidize palmitoyl carnitine in the presence of “classical” substrates glutamate, pyruvate, and succinate; and inclusion of fatty acids oxidation into the scheme of energy metabolism of astroglia removed many questions to astrocyte's functioning. It is time to change the current view on the neuronal energy metabolism as based almost exclusively on glucose.

## 19. Comment

Soon after we have submitted the paper of this review to the Biomed Research International, Schonfeld and Reiser have published a review, which was entitled: “Why does brain metabolism not favor burning of fatty acids to provide energy? Reflections on disadvantages of the use of free fatty acids as fuel for brain” [158]. The conclusions of the authors, which are reflected in the title, were quite opposite to the conclusions we present in the current review. Of course, it would be interesting and instructive to write a critical appraisal of the arguments presented by Schonfeld and Reiser and in the current review. But we think that, at the present, it is first necessary to provide the readers with our alternative point of view and evidences in support. As we mentioned in this review, some of the discrepancies stem from the differences in conditions and methods. For example, in one critical paper by Schönfeld et al., the authors did not observe reverse electron transport and ROS production during oxidation by rat heart mitochondria of the carnitine derivatives of fatty acids, which was observed (though also not high) with succinate as a substrate [157]. We have found that the authors used incubation medium, which contained 0.5 mM EDTA and rather high concentration of palmitoyl carnitine (0.5 mM). It is known that EDTA removes Mg^2+^ from the high affinity sites on mitochondria, and this causes an increase in the proton conductance of the inner membrane [11, 166]. As a result, the membrane potential drops by about 30–40 mV, which is enough to block RET [11]. In addition, in the rat heart mitochondria, oxidation of succinate is significantly inhibited by the endogenous oxaloacetate. The degree of inhibition does not depend on the presence of bovine serum albumin and varies between the animal species [11]. We hope that in the near future the discrepancies between the two groups will be resolved in the fruitful discussions for the benefit of the researchers working with mitochondria.

## Figures and Tables

**Figure 6:**
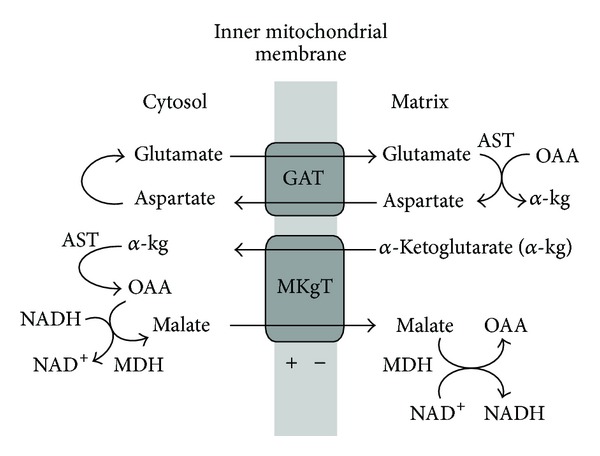
The malate-aspartate shuttle (MAS). The mitochondrial inner membrane is impermeable for NADH. In order to effectively utilize lactate for mitochondrial respiration, lactate must be converted to pyruvate in the reaction lactate + NAD^+^→ pyruvate + NADH. NADH is reoxidized by the MAS. The process is unidirectional because GAT (aralar) is electrogenic and the matrix NADH is rapidly oxidized by the respiratory chain.
